# Experimental demonstration of inverse-designed silicon integrated photonic power splitters

**DOI:** 10.1515/nanoph-2022-0443

**Published:** 2022-09-09

**Authors:** Junhyeong Kim, Jae-Yong Kim, Jinhyeong Yoon, Hyeonho Yoon, Hyo-Hoon Park, Hamza Kurt

**Affiliations:** School of Electrical Engineering, Korea Advanced Institute of Science and Technology, Daejeon 34141, Korea

**Keywords:** inverse design, MMI, nanophotonics, optical power splitter, particle swarm optimization, silicon photonics

## Abstract

The on-chip optical power splitter is a common and important device in photonic integrated circuits (PICs). To achieve a low insertion loss and high uniformity while splitting the guided light, multi-mode interferometer-based structures utilizing a self-imaging principle are widely used mainly in the form of a 1 × 2 configuration. Recently, an inverse design method for nanophotonic devices has emerged to overcome the limited capability of the conventional design methods and make it possible to explore the vast number of design parameters. Because of the non-intuitive shape of inverse-designed structures, they allow us to discover interesting and complex optical responses which are almost impossible to find with conventional design methods. Here, we report two kinds of inverse-designed 1 × 4 optical power splitters composed of silicon bars of different lengths, which are fabricated with a standard CMOS-compatible process. The particle swarm optimization method was used to minimize the insertion loss and divide the power evenly into each output port with finite-difference time-domain method simulation. The first optical power splitter has a compact size of 8.14 × 12 μm^2^ and the second optical power splitter has an even more compact size of 6.0 × 7.2 μm^2^. With the inverse designed structures, we fabricated the chip with a CMOS-compatible fabrication process. Experimental verification of the structures is provided and good agreement with the numerical results is obtained. The first 1 × 4 optical power splitter has a low insertion loss of less than 0.76 dB and uniformity of less than 0.84 dB, and the second more compact optical power splitter has a low insertion loss of less than 1.08 dB and uniformity of less than 0.81 dB. As the complexity of on-chip photonic systems has steadily increased, the inverse design of photonic structures holds great potential to be an essential part of advanced design tools.

## 1 Introduction

In recent years, nanophotonics has attracted great interest as a photonic integrated circuit (PIC) has huge advantages in terms of data rate, speed, transmission loss, and power consumption [1, 2]. In this process, increasing the performance of nanophotonic devices in such areas as optical power splitters (OPSs), grating couplers, and multiplexers is very important, while these devices directly determine the overall performance of a PIC. In particular, an optical power splitter is widely used in array devices like optical phased arrays [3–6] or photonic radar systems [7], photonic signal processing [8], programmable photonics [9], and quantum integrated circuits [10]. With the conventional design method, multimode interferometer (MMI) based optical power splitters [11–14], evanescent coupling-based optical power splitters [15–17], and photonic crystal-based optical power splitters [18–20] have already been proposed by various research groups. However, the conventional design method can be tested and designed with only a few design parameters, while it is strongly dependent on the designer’s intuition, experience, and numerical calculation. This is one of the reasons why most of the previous studies mainly focused on 1 × 2 power splitters and there are only limited studies on 1 × 4 optical power splitters. It is hard to design a structure with a small footprint and desired output response within the limited design area with the conventional design method.

Thanks to the advent of heuristic and intelligent algorithms such as particle swarm optimization (PSO) [21] and genetic algorithms (GA) [22], we can now examine various photonic structures that are difficult to explore with a forward design approach. As optimization with the algorithms finds the proper structures automatically with efficient optimization, this allows designers to reduce the time consumption of the design [23–25]. Very recently, it has been extended to a platform capable of inverse-designing the photonic structures based on artificial neural network, which allows the discovery of interesting structures and designs with more improved performances [26–30]. Some inverse-designed optical power splitters have been proposed with these advantages [31–37], and the majority of them had 1 × 2 configurations and Y-type topologies. In these structures, the presence of a bending region causes scattering and reflection losses. To have higher-order splitting, it is necessary to implement a cascaded version, but as a result, the photonic structure becomes lossy and bulky. Some of the inverse-designed structures have very small particles inside their structures, so they cannot be fabricated by the standard complementary metal-oxide-semiconductor (CMOS) process. This is one of the main considerations in applying inverse designs [38]. With this difficulty of fabrication, most of the studies proposed designs with simulation data only and without fabrication or experimental verification of the optical performance [31, 32, 34, 36].

In this paper, we propose two compact designs of a 1 × 4 optical power splitter which were inverse-designed using PSO. To the best of our knowledge, we firstly proposed inverse-designed 1 × 4 photonic power splitters in the SOI platform which is fabricated with conventional CMOS-compatible fabrication process. The design target of each structure has a figure of merit (FoM) that includes a low insertion loss and good uniformity with the compact size of the device. Our devices consist of input/output waveguides, grating couplers at the end, and tens of particles with different widths between the input and output waveguide. By optimizing the width and length of several particles, we expected to have some uniform power splitting effect with a low insertion loss. Compared to the subwavelength pixel-based structures or topological structures [34, 39], [40], [41], our design strategy has several advantages. Even though pixel-based structures or topological structures have larger degrees of freedom and have more chance to be well-optimized, the designed structures have very small features which cannot be easily fabricated. There might be some additional compensation to fabricate those topological structures which might give a different performance from the ideal design. Furthermore, optimization with a high degree of freedom is computationally demanding. Because there can be a large number of possible combinations of small particles, computation time will be too long even though optimization results may give superior performance. In our study, we proposed some constraints during the optimization process to reduce the demand for computation, which makes our design strategy efficient. Inverse-designed structures were verified with a finite-difference time-domain (FDTD) simulation and were fabricated with a CMOS-compatible fabrication process. The experimental results of the fabricated devices showed high performance in terms of insertion loss and uniformity and well-matched the simulation results. We discuss the details of the numerical and experimental findings in the following sections.

### 2 Design and simulation of 1 × 4 optical power splitter

**Figure 1:**
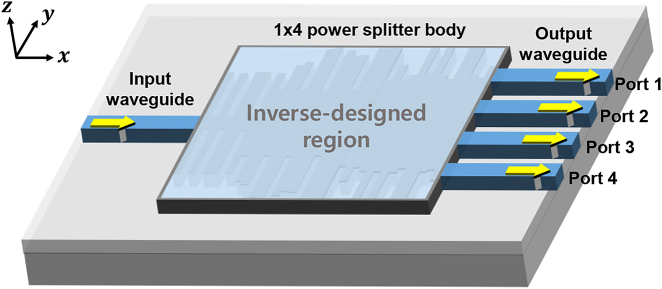
Schematic of the inverse-designed 1 × 4 optical power splitter on an SOI platform.

A schematic of the proposed optical power splitter is shown in Figure 1. Our design target was to divide the input light into four output channels equally, with an inverse design approach to the 1 × 4. The hardest part of this work was to keep both a low insertion loss and high uniformity. In the case of the 1 × 2 optical power splitter (OPS), we just focused on reducing the insertion loss, while keeping the structure symmetric, which guarantees high uniformity through the ports. However, in the case of the 1 × 4 OPS, focusing on the insertion loss do not guarantee the power uniformity of the structure. To achieve this scheme with a compact structure, the proper structure between input and output ports must be carefully designed. To design OPS with a low insertion loss and high uniformity, we needed to optimize the device parameters. Conventionally, researchers achieve this with numerical calculation and their intuition. For example, an MMI-based power splitter can be numerically designed based on the self-imaging principle. The concatenation of the single-mode input waveguide with the multimode waveguide section provides the interference of many higher-order modes. Consequently, constructive and destructive interferences are responsible for the appearance of intensity patterns. If the width and the length of the MMI region are correctly selected, then any number of uniform power splittings of the MMI can be obtained with a large footprint. In the case of a 1 × N MMI, the propagation constants of the excited modes approximately obey the following relation [42]:

$$\beta_{0}-\beta_{m}=\frac{m\left(m+2\right)\pi }{3L_{\pi }}$$

where *β*_0_ is the propagation constant of the fundamental mode in the MMI section and *m* is the mode number. With respect to the above equation, the beating length of the lowest-order modes *L_π_* and the length of the MMI for N-fold image *L*_MMI_ can be expressed as [42]:

$$L_{\pi }=\frac{\pi }{\beta_{0}-\beta_{1}}=4n_{\text{eff}}W_{e}^{2}/3\lambda$$

$$\;L_{\text{MMI}}=\frac{3rL_{\pi }}{4N}$$

where *n*_eff_ is the effective index in the multimode region, *W*_e_ is the effective width of the MMI, *λ* is the wavelength of the input light, and *N* is the number of output channels (the desired number of interference maxima). The coefficient *r* is an integer and can be set to 1.0 for the shortest MMI length. Even though the conventional MMI power splitter operates effectively, their overall dimensions are large, and considering many waveguide channels, one needs to have a multitude of cascaded MMIs to have 1 × 2^*n*^, where *n* ≥ 4. After deciding on the parameters using the above equation, optical simulation such as the finite-difference time-domain (FDTD) method can be used to verify its performance. Because of the limited parameter search among various structures, the performances of the devices are limited. An inverse design method has been proposed to break these limitations. With the desired optical response and limited design area, the optimization algorithm automatically finds the proper structure within the area.

Here, we used particle swarm optimization (PSO) to optimize the structure. PSO is a stochastic optimization algorithm proposed in 1995 [21]. It has been used for many kinds of optimization problems [43] and is also widely used to optimize nanophotonic structures [44–48]. While there are several evolutionary algorithms such as genetic algorithm (GA), differential evolution (DE), and PSO, we chose PSO over other algorithms for three reasons. First, PSO can reach a good solution without additional optimization (i.e. local search algorithm) compared to GA, which makes the algorithm effective [49]. Second, PSO has the advantage in terms of algorithmic simplicity compared to other algorithms [50]. Finally, PSO shows better performance than GA or DE with increasing problem dimensionality [51]. Starting from the initial position, each particle travels the parameter space to find the best position. In our optimization, considering both insertion loss and uniformity, the figure of merit (FoM) is set to be:

$$FoM=\alpha \left[1-\left(P_{1}+P_{2}\right)\right]+\beta \left|P_{1}-P_{2}\right|$$

where the *P*_1_ and *P*_2_ refer to the transmission of output port 1 and output port 2, respectively, and are not involved in FoM as *P*_1_/*P*_4_ and *P*_2_/*P*_3_ have the same value, because of the symmetry of the structure with respect to the centerline along the *x*-axis. While finding the best structure which has the minimum FoM with the PSO, the positions and velocities of the particles are defined according to the equation below:

$$\begin{array}{ll} v_{t} & =\omega v_{t-1}+c_{1}\eta_{1}\left(p_{\text{best},t-1}-x_{t-1}\right)\\ & \;+c_{2}\eta_{2}\left(g_{\text{best},t-1}-x_{t-1}\right) \end{array}$$

$$\;x_{t}=x_{t-1}+v_{t}$$

where *x* and *v* are the position and velocity of the particles. With iteration, *t*, *p*_best_ refers to the current best position and *g*_best_ refers to the global best position. *c*_1_ and *c*_2_ are the cognitive and social factors, and *η*_1_ and *η*_2_ are random coefficients between 0 and 1. *ω* is the inertial weight. Even though the transmission efficiency of a conventional MMI splitter is highly dependent on the boundary (geometry of the multimode region), a PSO-designed power splitter enables efficient operation under the irregular and highly corrugated boundaries transverse to the light propagation direction. The algorithm optimizes the lengths and widths of each silicon particle while evaluating the previously defined FoM.

**Figure 2:**
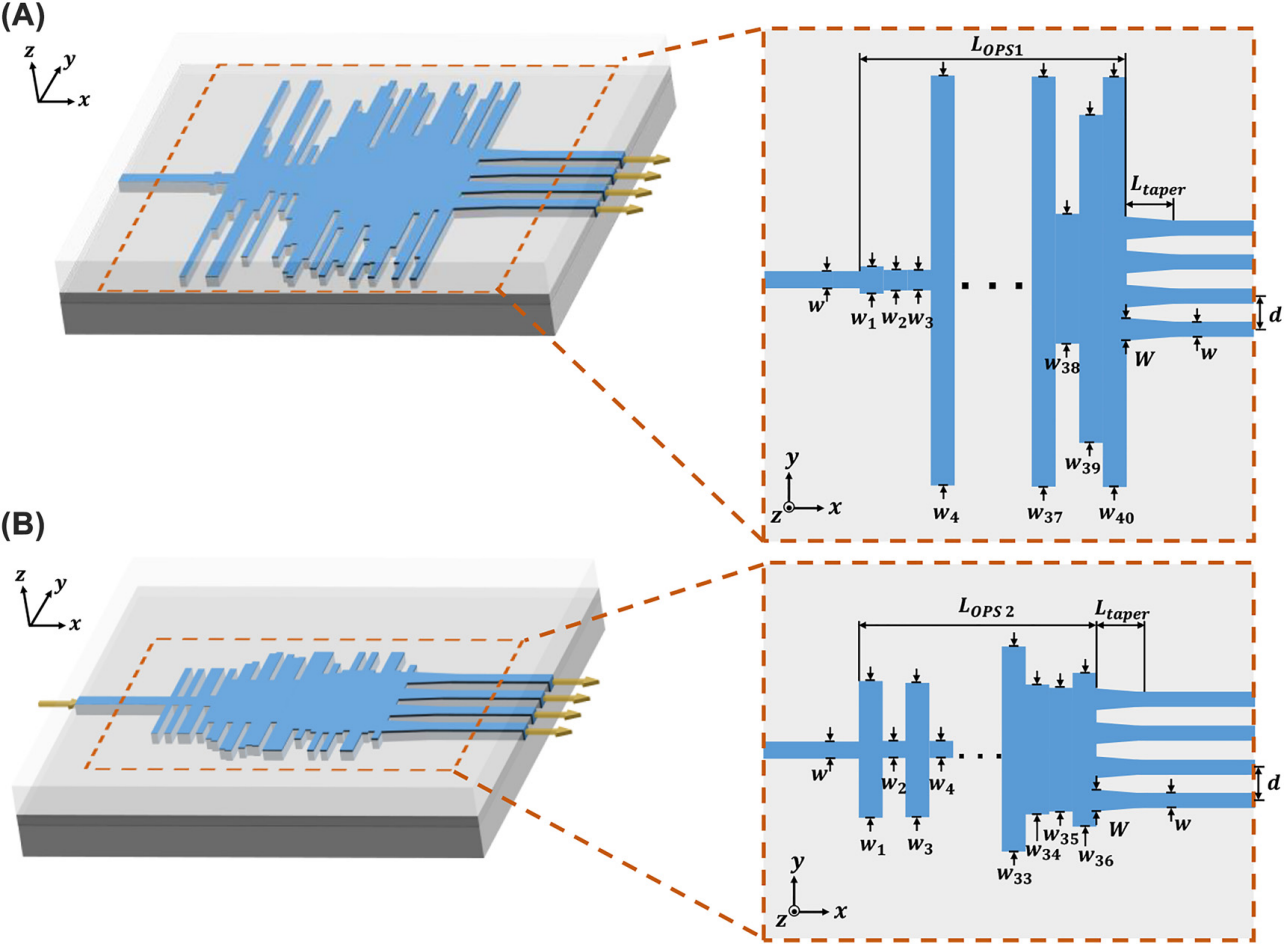
Schematic of the designs (A) OPS 1 and (B) OPS 2.

**Figure 3:**
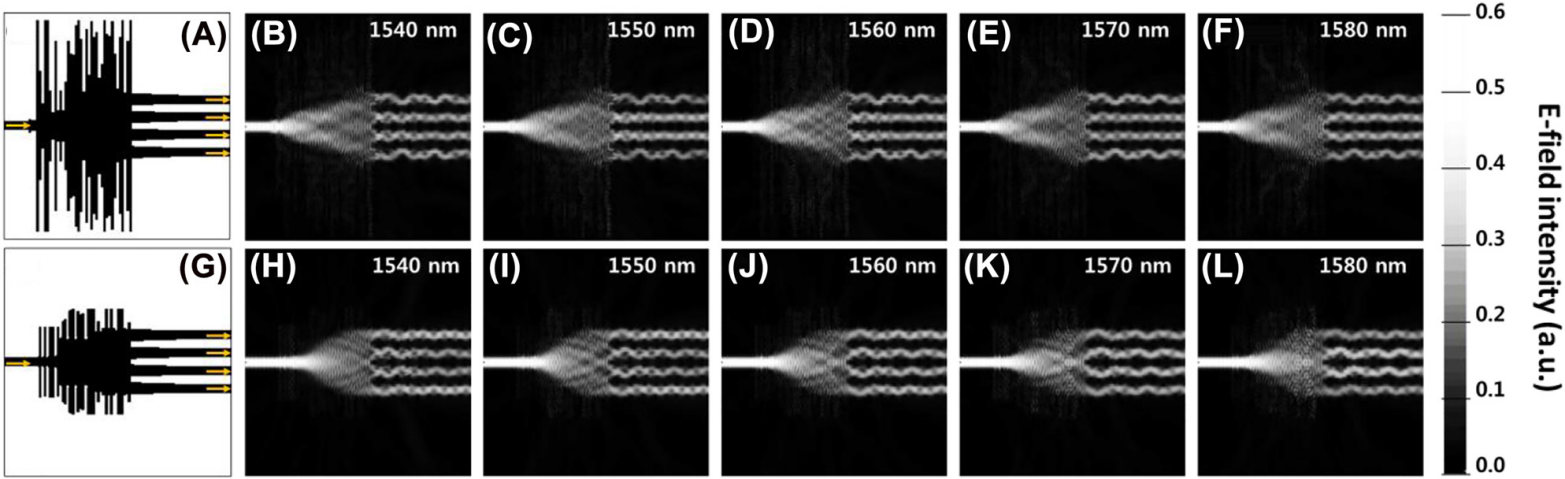
Designed structures of (A) OPS 1, and (G) OPS 2. Simulation results of the E-field distributions for the OPS 1 at different wavelengths, (B) 1540 nm, (C) 1550 nm, (D) 1560 nm, (E) 1570 nm, (F) 1580 nm and for the OPS 2 at (H) 1540 nm, (I) 1550 nm, (J) 1560 nm, (K) 1570 nm, (L) 1580 nm.

The detailed parameters of the OPS devices are shown in Figure 2. The C-band was chosen for the input light because it is widely used in optical communications. The OPS devices are designed on a silicon-on-insulator (SOI) platform, which has a silicon patterning layer height of 220 nm. The widths of the input and output waveguides are set to be 500 nm to excite the TE mode light into the single-mode waveguide, and tens of rectangular particles are located between the input and output waveguides. The centers of the four output channel waveguides are 1 μm apart (from the center of the waveguide to another) to avoid crosstalk between them. Each particle has an equal length of 150–210 nm and a width varying from 0.5 to 12 μm which is to be optimized by PSO. By optimizing the widths of each particle, we set the algorithm to maximize the transmission for the monitor at each output port while maintaining the uniformity between them. Here, we designed two different OPS devices. The first structure has a design area of 12 × 8.4 μm^2^ with 40 particles as illustrated in Figure 2A. In terms of reducing the footprint, we also tested with a smaller design area of 6 × 7.2 μm^2^ with 36 particles as illustrated in Figure 2B. After the optimization, we verified them with FDTD simulation. To satisfy the reliability of the fabrication process, we compensated for the widths and lengths of each particle. For instance, an ideally optimized particle width of 3.45632 μm was compensated for to 3.46 μm. A 2D simulation was performed because the 3D simulation is a time-consuming method, and it can be reduced to a 2D simulation using an effective index method [52, 53]. The simulation results of the E-field distributions are shown in Figure 3. For both structures, it is shown that the input light is successfully divided into four output channels for a band of 1540–1580 nm. Due to the increment of the wavelength of light, the power distributions through each channel slightly change. For example, for OPS2, the power is distributed slightly more at ports 1 and 4 at 1540 nm as shown in Figure 3H. When the wavelength of the light changes to 1580 nm, the power is distributed more at ports 2 and 3 as shown in Figure 3L. With these simulation results, we decided on each operation band for OPS 1 and OPS 2 where the power is evenly distributed for each port with a low insertion loss. As a result, OPS 1 and OPS 2 have operation bandwidths of 1555–1570 nm and 1545–1560 nm, respectively. A more detailed description of selecting the operation bandwidth for each device is discussed in Section 3.1.

## 3 Fabrication and experimental verification

### 3.1 CMOS compatible fabrication

**Figure 4:**
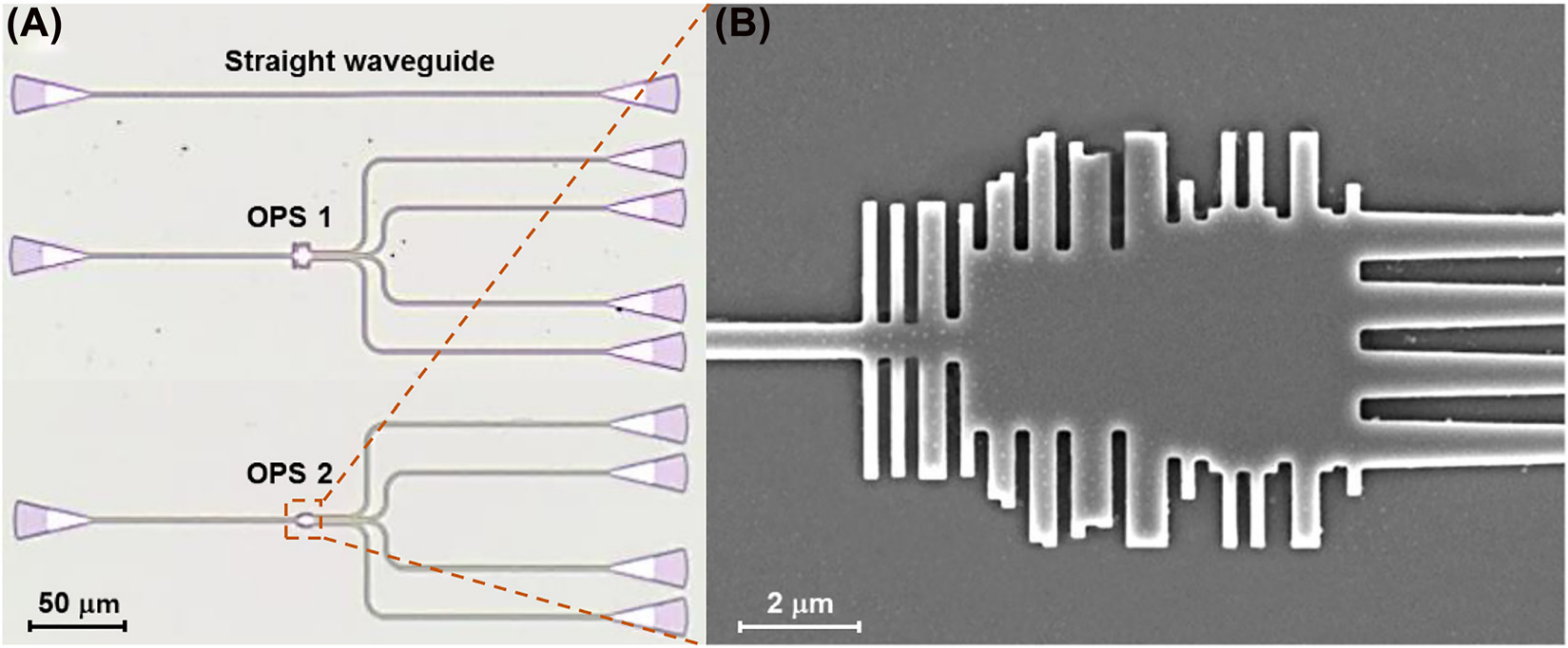
Images of the fabricated devices. (A) An optical microscopic image of the straight waveguide, OPS 1, and OPS 2, (B) SEM image of OPS 2 device.

Based on the optimized designs and the simulation described above, the device was fabricated with the SOI platform. We used the CMOS-compatible fabrication process provided by Applied Nanotools. The device had a silicon substrate of 725 μm, SiO_2_ box of 2 μm, silicon device layer of 0.22 μm, and top oxide cladding of 1.2 μm. A 100 KeV electron beam lithography technology was used to guarantee the reliability of the design, as the technique can fabricate the device with a minimum feature size of 60 nm. The waveguide was fully etched down to the buffer oxide using an e-beam mask material and an anisotropic ICP-RIE etching process. Optical microscopic image and scanning electron microscope (SEM) image of the fabricated device after the buffered oxide etching (BOE) process are shown in Figure 4.

### 3.2 Experimental verification

**Figure 5:**
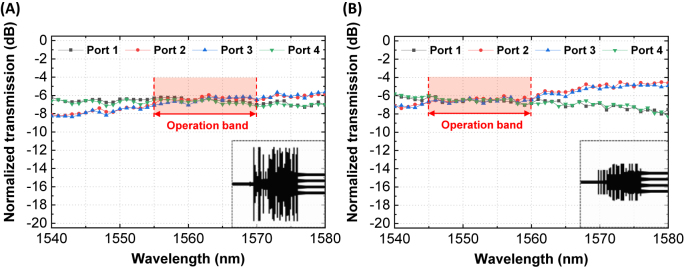
Measured transmission spectra of the fabricated 1 × 4 power splitters, (A) OPS 1 and (B) OPS 2.

To measure the transmission responses of the fabricated device, experimental verification is performed as follows. Input light from a tunable laser is guided to the input grating coupler through a single-mode optical fiber. Guided light travels through the device and reaches each of the four output waveguides. At each output, the light is radiated via grating couplers, captured by single-mode optical fiber, and launched into a photodetector. By sweeping the wavelength, we were able to obtain the transmission spectrum of each port. However, this measured transmission contains the loss from the input/output grating couplers and waveguides. To consider this additional loss, we measured the simple grating-to-grating straight waveguide structure under otherwise identical conditions. By subtracting this grating-to-grating loss, a reliable transmission spectrum of each port is obtained [13], and shown in Figure 5A and B. While the wavelength of the input light changes from 1540 to 1580 nm, the transmission spectrum of the OPS devices are given. We also highlighted the preferred operation band region for both devices (Figure 5A and B). The figures show that high uniformities (<1 dB) are secured within the operation band region for each device.

## 4 Discussion

### 4.1 Insertion loss and uniformity of the power splitter devices

**Figure 6:**
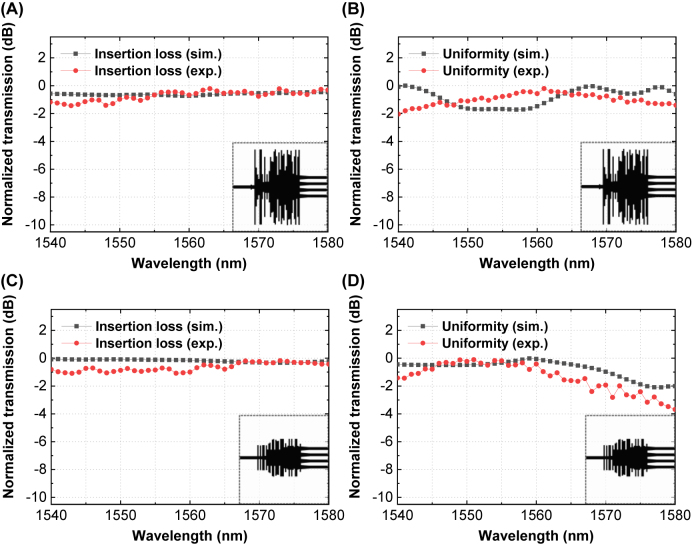
Comparison between simulation results and experimental results for the OPS 1 (A) insertion loss, (B) uniformity loss, and OPS 2 (C) insertion loss, and (D) uniformity loss.

With the simulation results and measured data, we obtained a comparison between them in terms of insertion loss and uniformity. The insertion losses (IL) and uniformities (U) are defined as in the equation below:

$$IL=-10\cdot \log\left[\left(P_{1}+P_{2}+P_{3}+P_{4}\right)/P_{in}\right]$$

$$U=-10\cdot \log\left[\min\left(P_{1},P_{2},P_{3},P_{4}\right)/\max\left(P_{1},P_{2},P_{3},P_{4}\right)\right]$$

where *P*_in_, *P*_1_, *P*_2_, *P*_3_ and *P*_4_ refer to the power transmission of the input port, output port 1, output port 2, output port 3, and output port 4, respectively. The comparison between the simulation results and measured data is shown in Figure 6. As the optimization method successfully achieved a low insertion loss as both OPS devices have a low insertion loss (<1 dB) within the bandwidth of 1540–1580 nm, the uniformity varies through the same bandwidth. Thus, we extracted a specific operation band region for each device to guarantee the uniform power distribution through each channel, which are 1555–1570 nm for OPS 1 and 1545–1560 nm for OPS 2. In Figure 6A and B, the insertion loss is compared for each OPS. For OPS 1, the measured insertion loss is less than 0.76 dB within the operation bandwidth. For OPS 2, the measured insertion loss is less than 1.08 dB within the operation bandwidth. In Figure 6B and D, the uniformity is compared for each OPS. For OPS 1, the measured uniformity is less than 0.84 dB within the operation bandwidth. For OPS 2, the measured uniformity is less than 0.81 dB within the operation bandwidth.

### 4.2 Analysis of the simulation result and the experimental result

In the numerical analysis, since the design region of the OPS 1 is larger than that of OPS 2, it is expected that OPS 1 would have a better performance than OPS 2. However, the simulation result shows that OPS 1 has an insertion loss through the bandwidth similar to OPS 2, and does not clearly perform better. In the experiment, we verified that OPS 1 performs better in terms of insertion loss compared to OPS 2. With these slight mismatches between the simulation and measurement, we discuss several observations in this section.

Firstly, in the inverse design process, the optimization method could not check all the possible combinations of the structures as it is a hugely time-consuming process. This may lead to the optimization result being trapped in the local minimum. We can increase the number of iterations/searches and change the coefficients carefully to avoid this problem, but then the optimization results are barely reasonable as they show a similar insertion loss on average. Moreover, we compensated for the parameters of the optimized structure to satisfy the resolution of the fabrication process, which may cause unexpected effects and small changes on the overall performance of the power splitter.

Secondly, depending on the fabrication conditions, the fabricated devices may have some slightly different parameters compared to the designed structures. For instance, electron scattering effects may occur when patterns are exposed to the mask in the e-beam lithography process [54]. In addition, misaligning between the mask and the etching process may create sidewall roughness in our device waveguide [55]. We speculate that the slightly different fabricated structure may allow us to get parameters that we never tested in our optimization process.

Despite considering these mismatches, the overall performances of the fabricated photonic power splitters are close to the simulation results in terms of the insertion loss and uniformity. The strongly corrugated side walls of the designed region produce large index modulation for the multimode section. Consequently, complex interference and scattering of light over a certain bandwidth give rise to the successful splitting of the incident power into four branches thanks to the design capability of inverse design methods.

## 5 Conclusions

In this study, we designed, simulated, and experimentally verified two 1 × 4 optical power splitters on an SOI platform. We used the PSO method to inverse design two 1 × 4 optical power splitters with low insertion loss and high uniformity. After optimization, the structure is compensated to fulfill the critical dimension and resolution of the lithography process during the fabrication. The designed structures were fabricated on SOI wafers using the e-beam lithography process and the performance of the fabricated devices were verified experimentally. The devices had compact sizes of 8.4×12 and 6.0×7.2 μm^2^, low insertion losses of 0.76 and 1.08 dB, and high uniformities of 0.84 and 0.81 dB. While our proposed 1×4 optical power splitters have a very low insertion loss and high uniformity with a small footprint, they can be widely applied to many photonic integrated circuits to replace conventional power splitters. Furthermore, with our proposed structures and design method, we can further improve several properties. The design approach allows us to target arbitrary power split ratios such as 1:1:1:1, 1:0.5:0.5:1, 0.5:1:1.5:2, etc. Besides, we may expand the structures with an increased number of output ports, and also different material platforms such as SiN can be investigated with the proposed approach.
